# Cardiac implantable electronic devices and radiation therapy: long-term outcomes

**DOI:** 10.1093/eurheartj/ehag314

**Published:** 2026-05-19

**Authors:** Mateusz Tajstra, Maciej Dyrbuś, Aleksandra Błachut, Joanna Machowicz, Tomasz Rutkowski, Jerzy Wydmański, Mariusz Gąsior, Sławomir Blamek

**Affiliations:** 3rd Department of Cardiology, School of Medical Sciences in Zabrze, Medical University of Silesia, Katowice, Poland; 3rd Department of Cardiology, Silesian Center for Heart Diseases in Zabrze, Skłodowskiej-Curie 9, 41-800 Zabrze, Poland; 3rd Department of Cardiology, School of Medical Sciences in Zabrze, Medical University of Silesia, Katowice, Poland; 3rd Department of Cardiology, Silesian Center for Heart Diseases in Zabrze, Skłodowskiej-Curie 9, 41-800 Zabrze, Poland; 3rd Department of Cardiology, School of Medical Sciences in Zabrze, Medical University of Silesia, Katowice, Poland; 3rd Department of Cardiology, Silesian Center for Heart Diseases in Zabrze, Skłodowskiej-Curie 9, 41-800 Zabrze, Poland; 3rd Department of Cardiology, School of Medical Sciences in Zabrze, Medical University of Silesia, Katowice, Poland; 3rd Department of Cardiology, Silesian Center for Heart Diseases in Zabrze, Skłodowskiej-Curie 9, 41-800 Zabrze, Poland; Department of Radiotherapy, Maria Skłodowska-Curie National Research Institute of Oncology, Gliwice Branch, Gliwice, Poland; Department of Radiotherapy, Maria Skłodowska-Curie National Research Institute of Oncology, Gliwice Branch, Gliwice, Poland; 3rd Department of Cardiology, School of Medical Sciences in Zabrze, Medical University of Silesia, Katowice, Poland; 3rd Department of Cardiology, Silesian Center for Heart Diseases in Zabrze, Skłodowskiej-Curie 9, 41-800 Zabrze, Poland; Department of Radiotherapy, Maria Skłodowska-Curie National Research Institute of Oncology, Gliwice Branch, Gliwice, Poland; Department and Clinic of Oncology, School of Medical Sciences in Zabrze, Medical University of Silesia, Katowice, Poland

## Introduction

The prevalence of cancer and the number of patients living with cardiac implantable electronic devices (CIEDs) continue to rise. Consequently, an increasing proportion of patients with CIEDs require radiation therapy (RT).^1–3^ Although RT is generally considered safe, concerns persist regarding device malfunction, particularly with high energies and dose *per* generator.^4,5^ Current European Society of Cardiology (ESC) Cardio-Oncology and national recommendations provide structured risk stratification and management pathways, but focus primarily on acute device safety.^6^ However, real-world data validating these recommendations remain limited, and long-term post-RT device outcomes have not been systematically evaluated.^7–9^ Given the stochastic nature of radiation effects, delayed CIED dysfunction may be a concern. In 2016, a dedicated cardio-oncology programme was established between a tertiary RT centre and a high-volume cardiac electrophysiology department.^10^ This analysis reports early- and long-term outcomes of patients managed within this framework.

## Methods

All consecutive patients with any CIED who underwent RT between January 2015 and January 2024 were included. Prior to RT, patients underwent joint evaluation by a radiation oncologist and a cardiologist experienced in CIEDs. RT assessment included beam energy, estimated dose to the device, and distance between the irradiated target and generator, while risk stratification followed ESC recommendations. Photon energies <10 MV were preferentially used, and efforts were made to limit generator dose to <2 Gy or <5 Gy whenever feasible. Device relocation was not routinely recommended in any case and was reserved for cases in which RT delivery was deemed impossible due to device proximity. Pacing dependence or ICD indication alone did not constitute an indication for relocation but influenced peri-procedural management.^5^

**Figure 1:**
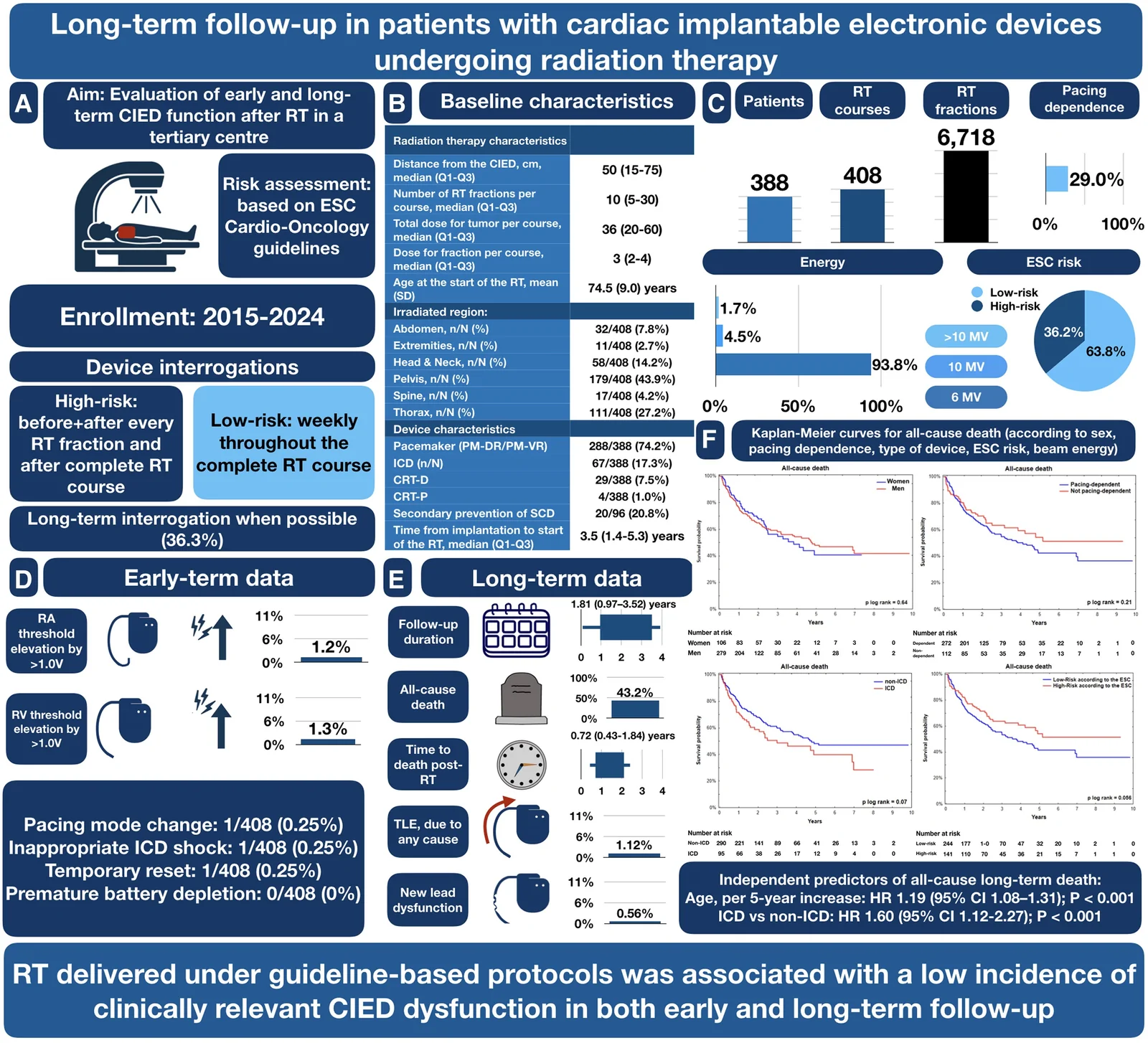
Study summary. (*Panel A*): Summary of the aims, and methods used in the study. Patients were recruited between 2015 and 2024, and all were stratified according to the ESC Cardio-Oncology guidelines. (*Panel B*): baseline patient, device, and RT characteristics. (*Panel C*): Graphical summary of the distribution of patients by the ESC risk category, pacing dependence, and beam energy. (*Panel D*): Early-term data indicate a low incidence of acute device-related adverse effects. (*Panel E*): Long-term data on the patient, and device-related factors, including follow-up duration, indicate a low incidence of long-term device-related events. (*Panel F*): Kaplan–Meier curves for all-cause mortality according to sex, device type, pacing dependence, and ESC guideline-derived RT-related CIED risk

All patients were assessed before and after each RT course. Moreover, patients with implantable cardioverter-defibrillators (ICDs), cardiac resynchronization therapy-defibrillators (CRT-Ds), or high-risk pacemakers (PMs) underwent interrogation before and after each RT fraction, while intermediate- and low-risk PM patients were assessed weekly following confirmation of safety during the first fraction (*Figure 1A*). Acute device-related adverse events were prospectively documented. For patients followed in the long-term in external clinics, device-related events (including generator replacement or lead extraction) were assessed using administrative records and structured telephone interview. Survival time, based on administrative records, was calculated from the date of RT initiation to death or censoring at the end of the study period (30 November 2024). Kaplan–Meier methods estimated time-specific mortality, and Cox regression identified mortality predictors. Long-term follow-up was defined as any device-related or clinical outcome after RT completion. All patients provided informed consent to participate in the study.

## Results

A total of 388 patients with CIEDs underwent 408 RT courses (*Figure 1B* and *C*); 15 patients (3.9%) received more than one course. Mean (SD) age at RT initiation was 74.5 (9.0) years, and median time from device implantation to RT was 3.5 years (IQR 1.4–5.3). PMs constituted 75.0% of devices, and 29.0% of patients were pacing-dependent. The pelvis was the most frequently irradiated region (43.9%), followed by the thorax (27.2%). According to ESC criteria, 36.8% of RT courses were classified as high risk. There were 7 RT courses (1.7%) using energies >10 MV. No RT plan required strategy modification (e.g. from radical to palliative therapy), dose reduction, or device relocation because of the CIED.

### Early device-related outcomes

Acute, relevant device malfunctions were rare (*Figure 1D*). Small increases in pacing thresholds (≥1.0 V at 0.4 ms) occurred in 1.2% of right atrial and 1.3% of right ventricular leads. Minor, clinically insignificant impedance fluctuations were observed in <25% of patients. One transient reset resolved without intervention. No permanent device failures occurred during RT.

### Long-term outcomes

Median follow-up duration was 1.81 years (IQR 0.97–3.52), as presented in *Figure 1E*. A total of 43.2% of patients died throughout the study period, and Kaplan–Meier-estimated 1-year all-cause mortality was 22.9% (95% CI 18.7%–27.1%). No statistically significant associations were observed between pacing dependence, sex, or ESC RT-related risk category, and mortality. Among 36.3% of patients with detailed device follow-up, no premature battery depletion or late device reset was identified. Twenty-seven elective secondary procedures (26 generator replacements and 1 upgrade) occurred at expected intervals. Late changes in lead parameters were uncommon and did not require lead revision or extraction. Two transvenous lead extractions occurred for indications unrelated to RT. Kaplan–Meier survival curves are shown in *Figure 1F*. In multivariable Cox regression analysis, increasing age (per 5-year increase) and device type (ICD vs non-ICD) were associated with all-cause long-term mortality.

## Discussion

This study provides the first long-term real-world evaluation of CIED performance following RT. Our findings confirm that photon-based RT delivered according to contemporary protocols is associated with a low incidence of acute and delayed device dysfunction. No significant associations were observed between pacing dependence, sex, ESC RT-related risk category, and mortality. These findings suggest that long-term prognosis may be driven by oncologic factors and comorbidity burden rather than RT-related device risk. ESC guideline-derived RT-related risk stratification estimates the risk of acute device malfunction, and thus, may not apply for mortality prediction post-RT. The predominance of non-thoracic irradiation (73%), low generator doses, and limited use of neutron-producing energies likely contributed to the low incidence of RT-related CIED events; however, these characteristics reflect current practice and indicate that most patients with CIEDs are low- or intermediate-risk. Although a substantial proportion of patients were followed externally post-RT, survival status was available for the entire cohort. While subclinical device abnormalities cannot be excluded in patients without direct interrogation, no signal suggesting premature CIED failure emerged.

### Limitations

This was a single-centre observational analysis, and results may not generalize to populations with higher proportions of neutron-generating therapies, while residual confounding cannot be excluded. Long-term device interrogation was available in 36.3% of patients, and cause-specific mortality could not be determined. Among survivors, 102 could not be reached despite contact attempts.

## Conclusions

In a large real-world cohort with extended follow-up, photon-based RT delivered under guideline-derived protocols was associated with a low incidence of clinically relevant acute or long-term CIED dysfunction. These findings support current risk-stratified management strategies and confirm the long-term safety of RT in patients with CIEDs.

## Declarations

### Data Availability

The dataset is available upon reasonable request from the corresponding author.
